# Assessing the Risk That *Phytophthora melonis* Can Develop a Point Mutation (V1109L) in CesA3 Conferring Resistance to Carboxylic Acid Amide Fungicides

**DOI:** 10.1371/journal.pone.0042069

**Published:** 2012-07-27

**Authors:** Lei Chen, Shusheng Zhu, Xiaohong Lu, Zhili Pang, Meng Cai, Xili Liu

**Affiliations:** 1 Department of Plant Pathology, College of Agriculture and Biotechnology, China Agricultural University, Beijing, China; 2 Key Laboratory of Agro-Biodiversity and Pest Management of Education Ministry of China, Yunnan Agricultural University, Kunming, Yunnan, China; Louisiana State University, United States of America

## Abstract

The risk that the plant pathogen *Phytophthora melonis* develops resistance to carboxylic acid amide (CAA) fungicides was determined by measuring baseline sensitivities of field isolates, generating resistant mutants, and measuring the fitness of the resistant mutants. The baseline sensitivities of 80 isolates to flumorph, dimethomorph and iprovalicarb were described by unimodal curves, with mean EC_50_ values of 0.986 (±0.245), 0.284 (±0.060) and 0.327 (±0.068) µg/ml, respectively. Seven isolates with different genetic background (as indicated by RAPD markers) were selected to generate CAA-resistance. Fifty-five resistant mutants were obtained from three out of seven isolates by spontaneous selection and UV-mutagenesis with frequencies of 1×10^−7^ and 1×10^−6^, respectively. CAA-resistance was stable for all mutants. The resistance factors of these mutants ranged from 7 to 601. The compound fitness index (CFI  =  mycelial growth × zoospore production × pathogenicity) was often lower for the CAA-resistant isolates than for wild-type isolates, suggesting that the risk of *P. melonis* developing resistance to CAA fungicides is low to moderate. Among the CAA-resistant isolates, a negative correlation between EC_50_ values was found for iprovalicarb vs. flumorph and for iprovalicarb vs. dimethomorph. Comparison of the full-length cellulose synthase 3 (CesA3) between wild-type and CAA-resistant isolates revealed only one point mutation at codon position 1109: a valine residue (codon GTG in wild-type isolates) was converted to leucine (codon CTG in resistant mutants). This represents a novel point mutation with respect to mutations in CesA3 conferring resistance to CAA fungicides. Based on this mutation, an efficient allelic-specific PCR (AS-PCR) method was developed for rapid detection of CAA-resistance in *P. melonis* populations.

## Introduction

The oomycete *Phytophthora melonis* Katsura, which is conspecific with *P. sinesis*, causes a severe disease of cucumber (*Cucumis sativus*) which has been reported in China, Japan, Egypt, Turkey, Korea, India and Iran [1]. In addition to cucumber, *P. melonis* infects other cucurbits including zucchini (*Cucurbita pepo* L.), hami melon (*Cucumis melo* L.), wax gourd (*Benincasa hispida* (Thunb.) Cogn.) [2]–[5], and pointed gourd (*Trichosanthes dioica* Roxb.) [6]. It also infects pistachio (*Pistacia vera* L) [7], causing blight, dieback, root rot, foot rot and crown rot. The use of resistant cultivars and chemical fungicides are two efficient control methods [2], [5], [8]. Phenylamides (e.g. metalaxyl) have been widely used for *P. melonis* disease control. However, metalaxyl-resistance of *P. melonis* has been reported in China [9]. Since the early 1980s, the efficacy of phenylamides has declined due to the emergence of resistant populations of oomycete pathogens in fields [10], [11].

The current study concerns resistance of *P. melonis* to the carboxylic acid amide (CAA) fungicides, which are divided into three different chemical groups based on differences in structure: the cinnamic acid amides (e.g., dimethomorph and flumorph), the valine amide carbamates (e.g., benthiavalicarb, benthiavalicarb-isopropyl and iprovalicarb) and the mandelic acid amides (e.g., mandipropamid) (FRAC Code List, www.frac.info). These fungicides are used to control the pathogens in the families Peronosporaceae (e.g., *Plasmopara viticola* and *Bremia lactucea*) and Pythiaceae (e.g., *Phytophthora* spp., but not *Pythium* spp.) [12]. All CAA fungicides strongly inhibit all asexual stages of susceptible pathogens but do not inhibit zoospore release and mobility [13]–[16]. Inhibition by CAA fungicides results from the interruption of cellulose biosynthesis and the disruption of cell wall structure [17].

istance to phenylamide fungicides, resistance to CAA fungicides is an important problem. Since dimethomorph’s introduction in the 1980s, CAA-resistant isolates of *P. viticola* have been detected in most areas of Europe (FRAC web). In China, flumorph-resistant isolates of *Pseudoperonospora cubensis* were obtained after successive applications of flumorph in a greenhouse [18]. *P. viticola* and *Ps. cubensis* are classified by FRAC as being at high risk to develop resistance to CAA fungicides, but *P. infestans* is considered to have a low risk of developing such resistance (FRAC pathogen risk list, www.frac.info). No resistant isolates of *P. infestans* have been detected in field since the introduction of CAA fungicides over 15 years ago (CAA Minutes 2010 final RG, www.frac.info). Using UV- and EMS- mutagenesis, researchers obtained stable CAA-resistant mutants of *P. infestans* only with difficulty [19]–[21] and an isolate of *P. capsici* failed to develop dimethomorph-resistance after repeated exposure to the fungicide [19]. However, *P. capsici* mutants with high CAA-resistance were obtained by mass selection from zoospores and oospores, and the resistance risk was considered low to moderate to CAAs in *P. capsici* [22], [23].

Cross-resistance among CAA fungicides has been reported in *P. viticola* [24] and *P. capsici* [22]. The resistance mechanism of some pathogens to CAA fungicides has been elucidated in recent reports. Amino acid substitutions at codon position 1105 (G1105V or G1105A) in cellulose synthase (CesA3) were responsible for resistance to the CAA fungicide mandipropamid in EMS-generated mutants of *P. infestans* [17]. Changes of G1105V or G1105W led to CAA-resistance in *Ps. cubensis* [25]. In *P. viticola*, changes of G1105S in PvCesA3 conferred CAA-resistance [26]. Based on the point mutation, a PCR-RFLP method has been developed for detecting CAA-resistant isolates in *P. viticola* populations [27].

The objectives of the present study were to (i) determine the baseline sensitivity of *P. melonis* to the CAA fungicides flumorph, dimethomorph and iprovalicarb; (ii) assess the risk of resistance to the three CAA fungicides; (iii) investigate the CAA-resistance mechanism in *P. melonis*; and (iv) develop a rapid and reliable method for detection of CAA-resistant isolates in populations of *P. melonis*.

## Results

### Baseline Sensitivity of *P. melonis* to Flumorph, Dimethomorph and Iprovalicarb

**Figure 1:**
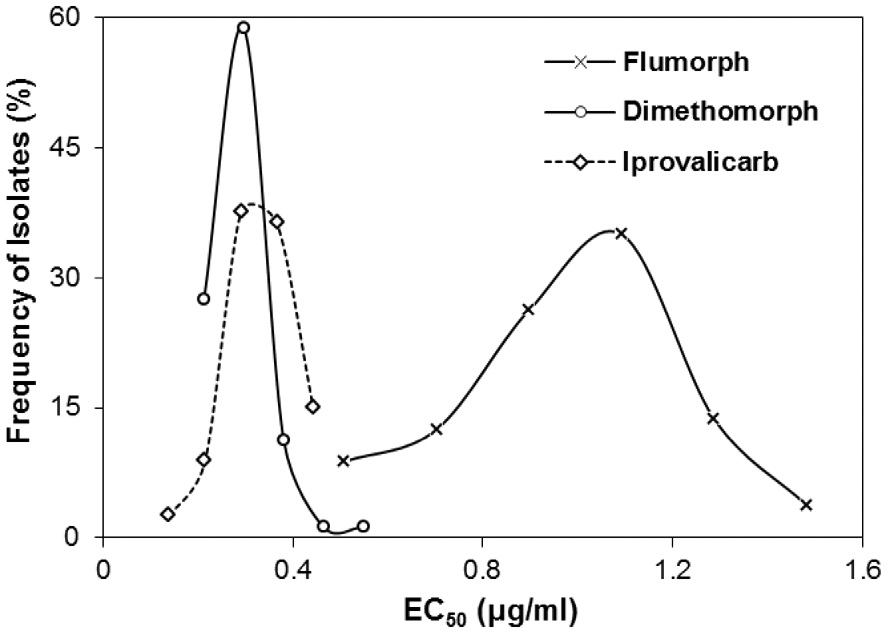
Frequency distributions of EC_50_ values (the effective concentration causing 50% inhibition of mycelial growth of *Phytophthora melonis*) for flumorph, dimethomorph and iprovalicarb. In total, 80 isolates of *P. melonis* were collected from areas never exposed to carboxylic acid amide fungicides.

For the 80 *P. melonis* isolates investigated, the frequency distribution of EC_50_ values for each of the three CAA fungicides were described by unimodal curves (Figure 1), indicating the absence of CAA-resistant subpopulations among these isolates. The mean and range of EC_50_ values were 0.986±0.245 µg/ml and 0.410–1.577 µg/ml for flumorph, 0.284±0.060 µg/ml and 0.171–0.590 µg/ml for dimethomorph, and 0.327±0.068 µg/ml and 0.100–0.482 µg/ml for iprovalicarb. The highest EC_50_ value was 3.45-, 3.85-, and 4.82-times greater than the smallest value for flumorph, dimethomorph and iprovalicarb, respectively.

### Development of CAA-resistant Isolates of *P. melonis in vitro*

#### Random amplified polymorphic DNA (RAPD)

**Figure 2:**
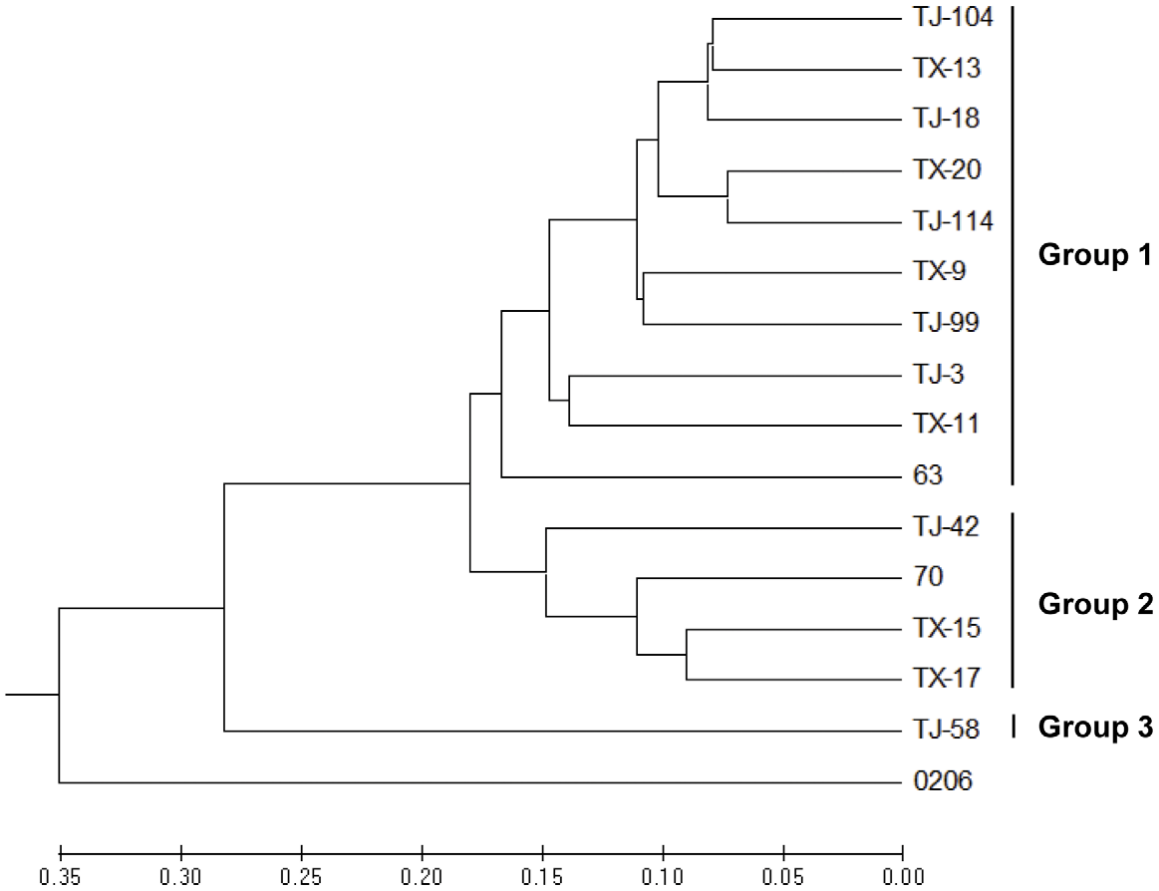
Genetic relationships among 15 isolates of *Phytophthora melonis*. The denrogram (UPGMA) shows the relationships among the isolates of *P. melonis* based on randomly amplified polymorphic DNA (RAPD) analysis with 16 decamer primers. Scale at the bottom depicts the genetic distance.

RAPD analysis was used to identify isolates with different genetic backgrounds; these isolates would be used as the parents for the generation of CAA-resistant mutants. Sixteen primers (Table 1) that produced easily recognizable and consistent banding patterns were used for RAPD analysis of 16 isolates from different geographic origins (Table 2). RAPD analysis using primer combinations clearly separated these isolates. A dendrogram based on UPGMA analysis indicated that the 15 isolates of *P. melonis* formed three major groups (Figure 2). One isolate of *P. drechsleri* was separated from *P. melonis*. RAPD groups were not related to the geographic origins or sensitivity to CAA fungicides of these isolates. Seven isolates (TJ-58, TX-11, TJ-99, TJ-104, TJ-114, 63 and 70) from different RAPD groups were selected for generation of CAA-resistant mutants.

**Table 1:** Nucleotide sequences and characteristics of primers used in this study.

Primers	Sequence (5′–3′)	Description	Source or reference
ABA3	AGTCAGCCAC	Primer for RAPD analysis	[42]
ABA7	GAAACGGGTG	Same as for ABA3	[42]
ABA9	GGGTAACGCC	Same as for ABA3	[42]
ABA10	GTGATCGCAG	Same as for ABA3	[42]
ABA13	CAGCACCCAC	Same as for ABA3	[42]
ABA17	GACCGCTTGT	Same as for ABA3	[42]
ABA18	AGGTGACCGT	Same as for ABA3	[42]
ABA20	GTTGCGATCC	Same as for ABA3	[42]
Y11	AGACGATGGG	Same as for ABA3	[43]
Y04	GGCTGCAATG	Same as for ABA3	[43]
OPG-16	AGCGTCCTCC	Same as for ABA3	[44]
OPS-14	AAAGGGGTCC	Same as for ABA3	[44]
OPX-12	TCGCCAGCCA	Same as for ABA3	[44]
OPG-11	TGCCCGTCGT	Same as for ABA3	[44]
OPG-14	GGATGAGACC	Same as for ABA3	[44]
OPG-15	ACTGGGACTC	Same as for ABA3	[44]
PmA3F1	TCTCGTGTCGGACGGACCAA	Primer for amplification and partial sequencingof *PmCesA3* gene	This study
PmA3S1	ATCATCGCGTGCTACCTGC	Sequencing primer for *PmCesA3* gene	This study
PmA3S2	CCGTCTTTGTTGTTGGCGGACTG	Same as for PmA3S1	This study
PmA3S3	TCGACGTACTCGATCGCCA	Same as for PmA3S1	This study
PmA3S4	TCACTACATGGAACCGGTGACG	Same as for PmA3S1	This study
PmA3S5	TGGACGGTGGAGGTCGTCAG	Same as for PmA3S1	This study
PmA3S6	AAGCCGTCGCTTGCGTTCC	Same as for PmA3S1	This study
PmA3R1	TCTGCATGTCCAGCCTTCC	Same as for PmA3F1	This study
PMF	ATCTACGCTCGCGGTACCAAG	Primer for rapid detection of resistance	This study
PMR1109A	CGAACACCACGATGTACACCAG	Same as for PMF	This study
PMR1109B	CGAACACCACGATGTACACCTG	Same as for PMF	This study
PMR1109C	CGAACACCACGATGTACACCCG	Same as for PMF	This study
PMR1109D	CGAACACCACGATGTACACCGG	Same as for PMF	This study

**Table 2:** Isolates of *Phytophthora melonis* used for RAPD analysis and their sensitivities to flumorph, dimethomorph and iprovalicarb.

Isolate	EC_50_ b (µg/ml) for the three fungicides			Origin
	Flumorph	Dimethomorph	Iprovalicarb	
TX-9	1.240	0.280	0.230	Xiqing 1^c^, Tianjin
TX-11	1.080	0.260	0.310	Xiqing 2, Tianjin
TX-13	1.193	0.280	0.330	Xiqing 3, Tianjin
TX-15	0.983	0.250	0.330	Xiqing 4, Tianjin
TX-17	1.161	0.319	0.340	Xiqing 5, Tianjin
TX-20	0.857	0.240	0.320	Xiqing 6, Tianjin
TJ-3	1.091	0.296	0.354	Hexi 1, Tianjin
TJ-18	1.182	0.281	0.311	Hexi 2, Tianjin
TJ-42	0.760	0.300	0.370	Hexi 3, Tianjin
TJ-58	1.010	0.360	0.240	Hexi 4, Tianjin
TJ-99	0.552	0.250	0.400	Nankai 1, Tianjin
TJ-104	0.775	0.270	0.360	Nankai 2, Tianjin
TJ-114	1.022	0.280	0.360	Nankai 3, Tianjin
63	0.440	0.360	0.290	UC Riverside, USA
70	1.410	0.590	0.370	UC Riverside, USA
0206^a^	0.710	0.680	0.420	Nanjing

a One isolate of *Phytophthora drechsleri* was used as an outgroup control.

b EC_50_ values, the effective concentration for causing 50% inhibition of mycelial growth inhibition of *P. melonis*.

c Number represents a different field in the same district.

#### Generation of CAA-resistant isolates

When the seven isolates were exposed to the CAA fungicides, spontaneous mutation resulted in five isolates with flumorph-resistance, one with dimethomorph resistance and three with iprovalicarb resistance. These nine isolates with spontaneous mutations were obtained from TJ-58, 63 and 70, and the survival frequency was approximately 1×10^−7^ for mutants exposed to the three fungicides (Table 3). No resistant isolates were derived from the other parent isolates. Following the UV treatment, however, the survival frequency was increased to approximately 1×10^−6^, and 19 flumorph-resistant, 17 dimethomorph-resistant and 10 iprovalicarb-resistant mutants were obtained from the parent isolates TJ-58, 63 and 70 (Table 3).

**Table 3:** Results of the experiments conducted to induce resistance against flumorph, dimethomorph, and iprovalicarb in *Phytophthora melonis*.

Parental isolates	Fungicides	Type of induction^a^	No. of mutants	Survival frequency^b^ (×10^−6^)	EC_50_ c (µg/ml)	RF^d^
TJ-58	Flumorph	SM	2	0.40	114∼151	113∼150
		UV	5	1.00	48∼155	133∼431
	Dimethomorph	SM	0	–	–	–
		UV	5	1.00	40∼151	111∼419
	Iprovalicarb	SM	1	0.25	101	421
		UV	2	0.40	46∼193	192∼804
63	Flumorph	SM	2	0.40	58∼159	132∼361
		UV	10	2.00	16∼174	35∼395
	Dimethomorph	SM	1	0.25	47	131
		UV	8	1.60	5∼194	14∼539
	Iprovalicarb	SM	2	0.40	74∼104	206∼289
		UV	3	0.60	43∼174	119∼483
70	Flumorph	SM	1	0.25	63	45
		UV	4	0.80	22∼57	16∼41
	Dimethomorph	SM	0	–	–	–
		UV	4	0.80	9∼76	15∼129
	Iprovalicarb	SM	0	–	–	–
		UV	5	1.00	8∼42	22∼114

a SM, spontaneous mutation. UV, UV-mutagenesis.

b Survival frequency, number of mutants/total number of zoospores used for mutant generation.

c EC_50_, the effective concentration for causing 50% inhibition of mycelial growth inhibition of *P. melonis*.

d Resistance factor  =  EC_50_ of resistant isolates at the 10^th^ transfer/EC_50_ of its parent.

### Characteristics of CAA-resistant Mutants

#### Resistance stability and resistance factor

After 10 transfers on non-amended medium, the mutant isolates grew as well on fungicide-amended medium as on non-amended medium, indicating that the resistance to CAA fungicides was stable. The level of resistance, as indicated by the resistance factor (RF  =  EC_50_ of mutant at the 10^th^ transfer/EC_50_ of its parent), ranged from 7 to 601 (Table 3).

#### Mycelial growth, sporulation and virulence *in vitro*

Compared to the mycelial growth of the corresponding parents (TJ-58, 63 and 70), mycelial growth was faster for some resistant isolates and slower for others. For example, the mycelial growth rate relative to the parent TJ-58 was significantly decreased for F58-1, F58-3, F58-4 and I58-2 (*p*<0.05), but significantly increased for D58-2 and I58-1 (*p*<0.05) on the non-amended medium (Table 4). Virulence also increased or decreased, depending on the resistant isolate. Lesions were significantly larger for the resistant isolates D58-3, D58-2, I58-1, I58-2 and F58-1 than for their parent TJ-58 (*p*<0.05) (Table 4). The resistant isolates F58-3, D58-3, D58-5 and I58-1, however, produced fewer zoospores than the wild-type isolate (Table 4). A compound fitness index (CFI) was calculated: CFI  =  *in vitro* mycelia growth × zoospore production × lesion area on cucumber leaves. CFI values of resistant isolates were significantly lower for two of nine mutants derived from parent isolate TJ-58, for five of nine mutants derived from parent isolate 63, and all eight isolates derived from parent isolate 70 (Table 4). CFI values were never significantly greater for the mutants than for the parents and were frequently lower but without statistical significance.

**Table 4:** Fitness of CAA-resistant and -sensitive isolates of *Phytophthora melonis in vitro*.

Isolates a	Mycelial growth (mm)^b^	Zoospore production (×10^5^/cm^2^)	Lesion area on cucumber leaves (mm^2^)	CFI^c^ (×10^5^)
**TJ-58**	77 c	1.53 a	370 d	43566 ab
F58-1	73 e	1.30 abcd	398 bc	37728 bc
F58-3	72 e	1.22 bcd	380 cd	33684 c
F58-4	74 de	1.44 ab	383 cd	40980 abc
D58-2	81 a	1.41 abc	420 ab	47483 a
D58-3	76 cd	1.20 bcd	425 a	38712 abc
D58-5	77 c	1.16 cd	383 cd	34205 c
I58-1	80 ab	1.13 d	420 ab	37743 bc
I58-2	73 e	1.28 abcd	398 bc	36977 bc
I58-3	78 bc	1.35 abcd	368 d	38660 abc
**63**	78 c	0.76 a	555 c	32988 ab
F63-1	73 f	0.42 c	532 c	16296 e
F63-3	76 de	0.48 bc	473 d	17381 e
F63-5	75 e	0.75 a	476 d	27014 abc
D63-1	76 de	0.42 c	462 d	14893 e
D63-2	77 de	0.53 bc	457 d	18652 de
D63-8	81 b	0.51 bc	476 d	19648 cde
I63-2	83 a	0.39 c	583 b	18784 de
I63-5	77 cd	0.62 ab	542 c	25872 bcd
I63-9	72 f	0.76 a	621 a	34020 a
**70**	87 c	0.87 a	575 d	43719 a
F70-1	82 e	0.74 b	497 e	30125 cd
F70-5	91 a	0.58 de	619 bc	32809 bc
F70-11	83 e	0.70 bc	561 d	32795 bc
D70-1	87 cd	0.53 e	639 b	29280 cd
D70-5	79 f	0.68 bc	681 a	36690 b
I70-1	89 b	0.52 e	567 d	26310 d
I70-5	85 d	0.53 e	564 d	25505 d
I70-9	87 cd	0.63 cd	607 c	33155 bc

a Isolates in bold font are parents of the resistant isolates listed under them in regular font. Isolates starting with the letter F, D, and I, are flumorph-resistant mutants, dimethomorph-resistant mutants, and iprovalicarb-resistant mutants, respectively.

b For each parent and its resistant progeny, means followed by same letters are not significantly different according to Fisher’s least significance difference (α = 0.05).

c CFI (compound fitness index)  =  mycelial growth × zoospore production × lesion area on cucumber leaves.

#### Cross-resistance

**Figure 3:**
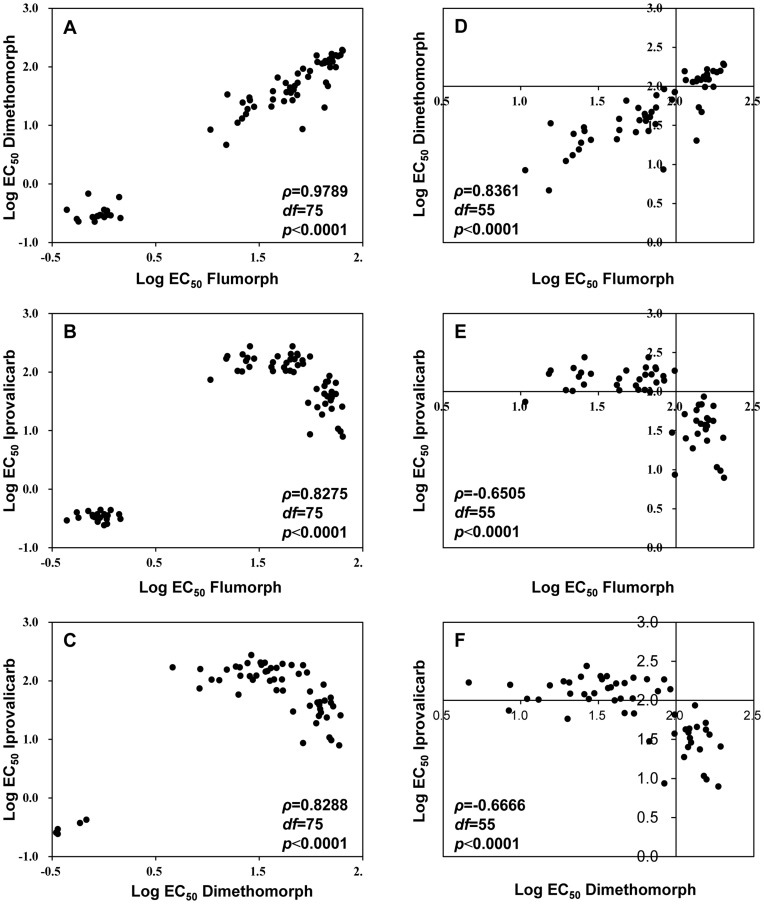
Cross-resistance among flumorph, dimethomorph and iprovalicarb. Log-transformed EC_50_ values (the effective concentration for causing 50% inhibition of mycelial growth inhibition of *Phytophthora melonis*) for isolates of *P. melonis* were compared among the three carboxylic acid amide fungicides using Spearman’s rank correlation coefficients. (A), (B), and (C) indicate positive cross-resistance among flumorph, dimethomorph, and iprovalicarb; (D-F) include only the higher EC_50_ values from (A-C), i.e., EC_50_ values from CAA-resistant isolates. (D) reveals a positive correlation between the EC_50_ values for dimethomorph and flumorph among CAA-resistant isolates, while (E) and (F) reveals a negative correlation between iprovalicarb and flumorph and between iprovalicarb and dimethomorph among CAA-resistant isolates.

There was a high level of cross-resistance among all three CAA fungicides: the values of Spearman’s rho (*ρ*) were all >0.8000 (*p*<0.0001) (Figure 3 A to C). Examination of the EC_50_ values (those for CAA-resistant isolates and clustered on the right side of Figure 3 A, B and C) once again indicated a positive correlation between resistance to dimethomorph and flumorph (Figure 3 D), but a negative correlation between resistance to iprovalicarb and flumorph (Figure 3 E) and between resistance to iprovalicarb and dimethomorph (Figure 3 F). No cross-resistance was detected between CAA fungicides and non-CAA fungicides such as metalaxyl, cymoxanil, azoxystrobin and cyazofamid (*p*>0.05) (data not shown).

### Analysis of the *CesA3* Gene in *P. melonis*

**Figure 4:**
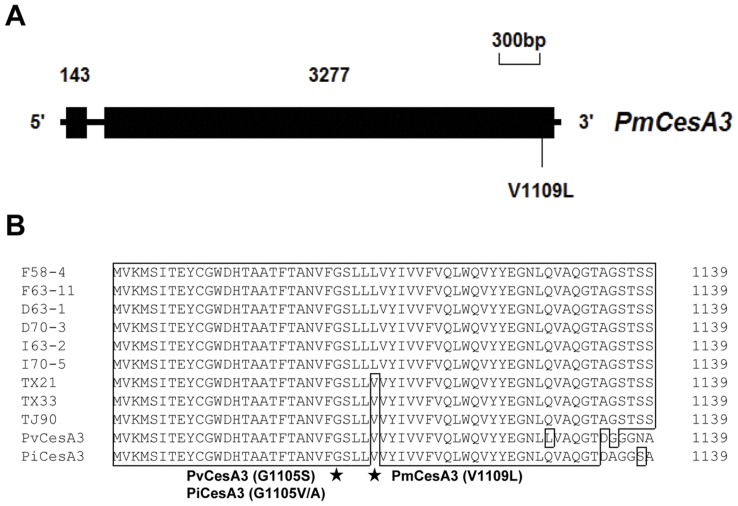
Structure and site of mutation in the *PmCesA3* gene associated with carboxylic acid amide (CAA) fungicide resistance. (A) Intron/exon structure of the *PmCesA3* gene. Numbers represent the size in base pairs. Point mutations in CAA-resistant mutants and the predicted amino acid substitution in the mutant gene products are indicated. (B) Alignment of partial amino acid sequences of CesA3 in *P. melonis* (PmCesA3), *P. infestans* (PiCesA3), and *P. viticola* (PvCesA3). TJ-90, TX-21, and TX-33 were wild-type isolates. D63-1 and D70-3 were dimethomorph-resistant mutants. F58-4 and F63-11 were flumorph-resistant mutants. I63-2 and I70-5 were iprovalicarb-resistant mutants. Mutations in CAA-resistant mutants of *P. infestans*, *P. viticola* and *P. melonis* are indicated by asterisks.

The full-length of *PmCesA3* gene contained 3550 bp, with one intron of 130-bp located after nucleotide 143 (Figure 4 A). The *PmCesA3* gene coded for a polypeptide chain of 1139 amino-acids and had a predicted molecular weight of 126.5 kDa. The analysis of identities between the PmCesA3 amino acid sequence and those of the closest organisms found in the NCBI GenBank database revealed that homologies were higher with the CesA3 in oomycetes than with CesA3 in *Arabidopsis thaliana* (Table 5). Compared to the CesA3 in sensitive isolates, only one amino-acid substitutions was detected in the CesA3 in the CAA-resistant isolates: this substitution (a GTG to CTG mutation) occurred at codon 1109 and resulted in the replacement of a valine residue with a leucine residue (Figure 4B).

**Table 5:** Predicted amino acid sequence identities (%) among known CesA3s from four *Phytophthora* species, *Plasmopara viticola*, and *Arabidopsis thaliana.*

	PmCesA3	PiCesA3	PrCesA3	PvCesA3	AtCesA3
PmCesA3	100	–	–	–	–
PiCesA3	82	100	–	–	–
PrCesA3	95	95	100	–	–
PvCesA3	81	95	94	100	–
AtCesA3	14	16	15	16	100

Values indicate identity expressed as a percentage.

### AS-PCR for Rapid Detection of CAA-resistant Isolates of *P. melonis*

**Figure 5:**
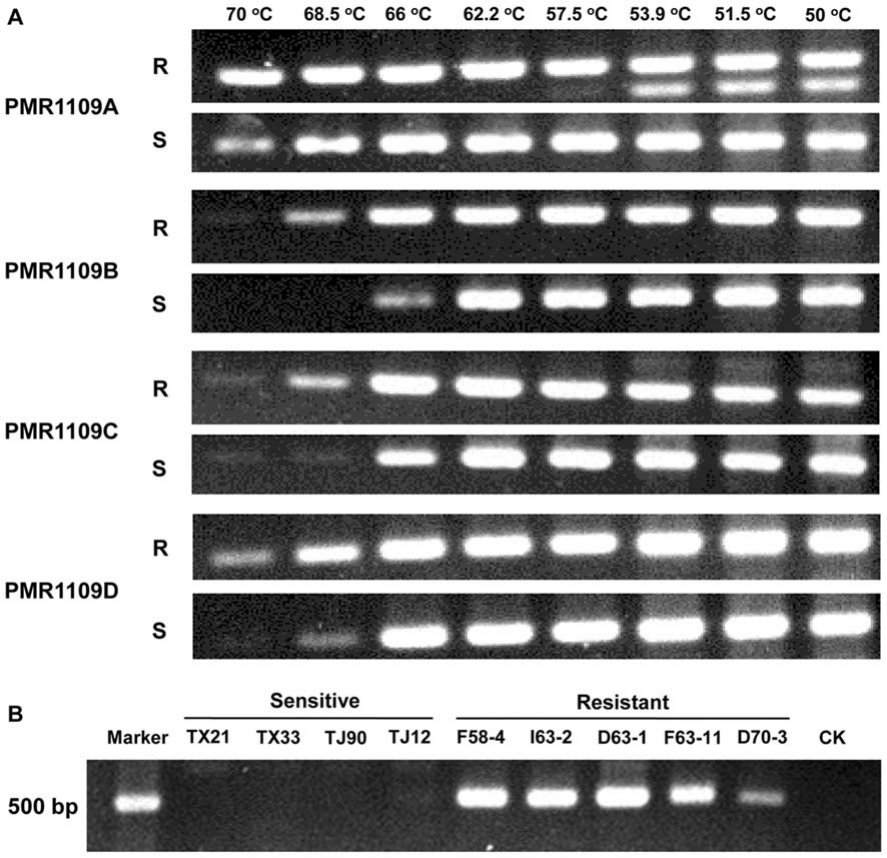
Specificity of four allele-specific PCR primer pairs for the detection of carboxylic acid amide (CAA)-resistant isolates of *Phytophthora melonis*. (A) Specificity of the four primer pairs for the CAA-sensitive isolate TJ-58 (S) and the CAA-resistant isolate F58-4 (R) at gradient annealing temperatures. (B) Specificity of primer pair (PMF + PMR1109B) for four CAA-sensitive and five CAA-resistant isolates at 68.5°C.

Four pairs of allele-specific primers, designed according to the single mutation in the *PmCesA3* gene, were used for PCR with DNA template from CAA-resistant and -sensitive isolates. Using the primer pair PMR1109A + PMF, a 500-bp fragment was amplified at different annealing temperatures whether the template DNA was from resistant or sensitive isolates (Figure 5A), indicating that primers designed by the traditional method could not discriminate between sensitive and resistant alleles. The introduction of an artificial mismatch base at the second nucleotide at the 3′-end of the primers improved specificity at various annealing temperatures (Figure 5 A). As the annealing temperature increased, the reverse primer with artificial mismatch ‘T’ at the second nucleotide showed more specificity than the primers with mismatch ‘C’ or ‘G’. At the annealing temperature of 68.5°C, the primer PMR1109B was optimal for distinguishing the mutation at codon 1109. With the primer pairs PMF + PMR1109B, the 500-bp fragment was amplified from CAA-resistant isolates F58-4, I63-2, D63-1, F63-11 and D70-3 but not from CAA-sensitive isolates TX21, TX33, TJ90 and TJ12 (Figure 5 B).

## Discussion

The sensitivity of 80 *P. melonis* isolates (collected from 13 fields in China) to the CAA fungicides flumorph, dimethomorph and iprovalicarb was determined by measuring EC_50_ values. The frequency distributions of the EC_50_ values were described as unimodal curves with a narrow range for each fungicide, indicating the absence of CAA-resistant subpopulations among the 80 isolates. Therefore, these results can be used as baselines for tracking future sensitivity shifts of *P. melonis* populations to these three CAA fungicides. Mycelial growth was inhibited more strongly by dimethomorph than by iprovalicarb or flumorph. Similar results were reported for *P. capsici* [22], [23], [28], *Bremia lactucae* [29], *P. infestans* [21], [30] and *Peronophythora litchi* [15], indicating that dimethomorph is generally more effective than iprovalicarb or flumorph for control of oomycete plant pathogens.

Although RAPD analysis revealed a high degree of genetic diversity in *P. melonis* collected from different geographical regions, the groups defined by RAPD markers did not share CAA-sensitivity. A likely reason for this lack of correlation is that RAPD markers could not reflected the defined loci responding to sensitivity to fungicides [31]. The RAPD results, however, made it possible to select isolates with different genetic backgrounds for resistance generation.

Isolates with resistance to CAA fungicides were generated from three of the seven isolates used, suggesting that the risk of *P. melonis* resistance to CAA fungicides may be associated with an isolate’s genetic background. This would explain why dimethomorph-resistant mutants of *P. capsici* could not be obtained from only one isolate by taming [21], but why CAA-resistance could be obtained by mass selection from zoospores and sexual progeny [22], [23].

The risk of fungicide resistance also depends on the pathogen species and its biological characteristics. Based on disease cycles, dispersal ability, frequency of sexual recombination and the competitive ability, *P. viticola* and *Ps. cubensis* have been considered high risk pathogens, while *P. infestans, P. capsici* and *P. melonis* have been considered low risk pathogens (FRAC, www.frac.info). Assessments of the risk of fungicide resistance are also based on field observations. Thus, CAA-resistant isolates that are stable and competitive have been detected among field populations of *P. viticola* [24] and *Ps. cubensis* [18] but not among field populations of *P. infestans* [20] (FRAC, www.frac.info), indicating a high risk of resistance to CAAs in *P. viticola* and *Ps. cubensis* but a low risk in *P. infestans*. Until now, no CAA-resistant isolates of *P. capsici* have been reported in the field, but *P. capsici* mutants with high CAA-resistance were obtained by mass selection from zoospores and oospores, and the risk of resistance to CAAs was considered low to moderate in *P. capsici* [22], [23]. For *P. melonis* in the current study, CAA-resistant mutants were generated *in vitro* with a frequency of 1×10^−7^ by spontaneous selection and 1×10^−6^ by UV-mutagenesis of zoospores. That the frequency was higher with UV-mutagenesis than with spontaneous selection suggests that UV radiation can increase the probability of CAA fungicide resistance in *P. melonis*. The CFIs (compound fitness indices) were often lower for the CAA-resistant isolates than the wild-type isolates, indicating that CAA-resistance in this study was generally associated with reduced fitness. This supports our inference that the risk of resistance to CAA fungicides in *P. melonis* is low to moderate.

Mutants resistant to one of the CAA fungicides in the current study were resistant to other CAA fungicides but not non-CAA fungicides, indicating that there was cross-resistance among flumorph, dimethomorph and iprovalicarb but not between the CAA and non-CAA fungicides. Similar results have been reported for *P. viticola* [24], *Ps. cubensis* [18] and *P. capsici* [22], [23], [32]. Although the cross-resistance suggests that the CAA-resistant isolates have a similar resistance mechanism, the negative correlation between higher EC_50_ values for iprovalicarb and flumorph and between higher EC_50_ values for iprovalicarb and dimethomorph but not between those for flumorph and dimethomorph suggests that the resistance mechanism may differ somewhat between the cinnamic acid amides (dimethomorph and flumorph) and the valine amide carbamates (iprovalicarb).

We amplified and sequenced the *CesA3* gene of *P. melonis*. Analysis of the CesA3 amino acid sequence revealed that the wild-type and CAA-resistant isolates of *P. melonis* differed only in the V1109L substitution (Figure 5B). Previous studies reported that resistance to CAA fungicides was conferred by G1105V or G1105A substitution in CesA3 of *P. infestans* [17], G1105S in CesA3 of *P. viticola* [26] and G1105V or G1105W in CesA3 of *Ps. cubensis* [25]. The substitution of V1109L in PmCesA3 would therefore represent a novel mutation causing resistance to CAA fungicides. The finding of only one mutation and the detailed cross-resistance results suggest that other genes might also be involved in CAA resistance. In addition, CAA resistance was considered to be controlled by a recessive gene in *P. infestans* [17] and *P. viticola* [26], but by two dominant genes in *P. capsici* [23]. In this study, we did not find any CAA-resistant isolates with a heterozygous mutation at codon position 1109 on PmCesA3, suggesting that CAA resistance in *P. melonis* may also be controlled by a recessive gene(s). Confirming this will require further genetic experiments, but genetic manipulation of *P. melonis* is difficult because it is homothallic.

Several methods such as AS-PCR and PCR-RFLP have been developed for detecting isolates with mutations associated with fungicides resistance [33]. A recent study described a PCR-RFLP method that rapidly detects CAA resistance in *P. viticola* populations [27]. In our study, AS-PCR primers were designed (based on the mutation of V1109L); these primers effectively identified CAA resistance in *P. melonis.* Compared with the traditional AS-PCR primers, the new reverse primer contained an additional mismatch at the second nucleotide of the 3′-end; the introduction of this mismatch was previously reported to increase specificity of the allele-specific primer [34]–[36]. In our trial, the mismatch nucleotide ‘T’ was more optimal than the mismatch nucleotides of ‘C’ and ‘G’. However, different mismatches can increase or decrease the specificity of the primer, indicating that the most suitable mismatch must be tested in different cases [37]. The AS-PCR primers described here will be useful for detecting CAA-resistant isolates of *P. melonis* from field populations.

## Materials and Methods

### Isolates and Culture Conditions

Roots and stems of cucumber (*Cucumis sativus* Linn.) with typical signs and symptoms of infection by *P. melonis* were collected from 13 fields in Xiqing, Hexi and Nankai districts in Tianjin of China in 2005, where CAA fungicides had never been used. Tissue plugs were cut from the margin of lesions on stems and roots. The plugs were disinfested for 3 min in 0.5% (vol/vol) NaClO. After being rinsed three times with sterile water, these plugs were placed on white kidney bean agar (WKB) (60 g of white kidney bean, 7 g of agar and distilled water up to 1 liter) plates amended with 50 µg/ml of ampicillin (98% a.i., Tuoyingfang Biotech Co., Ltd., Beijing), 50 µg/ml of rifampicin (98% a.i., Tuoyingfang Biotech Co., Ltd., Beijing) and 50 µg/ml of pentachloronitrobenzene (PCNB) (40% a.i., Sanli Chemical Industry Co., Ltd., Shanxi, China). After the cultures had been incubated at 25°C in darkness for 5 d, mycelial plugs were cut from margin of the culture and transferred to a new WKB agar plate. In total, 80 isolates of *P. melonis* were obtained. The isolates were identified using specific primers and morphology as described previously [1], [38].

For acquisition of single-zoospore isolates, a zoospore suspension was prepared by placing mycelial plugs into sterile soil extract (10 g of soil per liter of water). After incubation under light at 25°C for 72 h, sporangia formed. Following incubation at 4°C for 1 h and at 25°C for 40 min, zoospores were discharged from sporangia. A 0.2-ml volume of the zoospore suspension was placed on water-agar plate at 25°C. After 12 h, single germinated zoospores and associated agar were transferred to fresh WKB agar plate. Two single-zoospore isolates of *P. melonis* (68 and 70) were kindly provided by Dr. Michael D. Coffey (University of California, Riverside), and one single-zoospore isolate of *P. drechsleri* (0206) was kindly provided by Dr. Zheng Xiaobo (Nanjing Agricultural University). These three isolates were also single-zoospore cultures. All isolates were maintained on WKB agar medium. For long-term storage, each culture was transferred to WKB agar slants, covered with sterile mineral oil, and stored at room temperature.

### Fungicides

The following technical-grade fungicides were individually dissolved in dimethyl sulfoxide (DMSO) to prepare stock solutions (1×10^4^ µg/ml) and stored at 4°C in the dark: flumorph (96% a.i., Research Institute of Chemical Industry, Shenyang, China), dimethomorph (95% a.i., Frey Agrochemicals Ltd), iprovalicarb (98% a.i.; Sigma-Aldrich Shanghai Trading Co. Ltd), metalaxyl (97% a.i., Agrolex P. Ltd., Beijing), azoxystrobin (96% a.i., Syngenta Biotechnology Co. Ltd., Shanghai, China), cyazofamid (96% a.i., Sigma-Aldrich Shanghai Trading Co. Ltd) and cymoxanil (98% a.i., Xinyi Agrochemicals Company, Jiangsu, China). The final concentration of DMSO in the WKB agar medium was adjusted to 0.1% (vol/vol) throughout this study. WKB agar plates amended with fungicides were prepared by adding the same volume of serially diluted solutions to the molten agar medium at ≈50°C. WKB agar medium without fungicide but with the same volume of DMSO was used as a control.

### Baseline Sensitivities of *P. melonis* to Flumorph, Dimethomorph and Iprovalicarb

The sensitivities of 80 *P. melonis* isolates to the CAA fungicides flumorph, dimethomorph and iprovalicarb were determined by measuring mycelium growth on fungicide-amended medium. Fresh mycelial plugs (5 mm in diameter) were cut from the edge of an actively growing colony and placed face up in the center of WKB agar medium plates, which were amended with flumorph (0, 0.50, 0.70, 0.90, 1.00, 1.25, 1.50 µg/ml), dimethomorph (0, 0.10, 0.15, 0.20, 0.25, 0.30, 0.35 µg/ml) or iprovalicarb (0, 0.20, 0.25, 0.30, 0.35, 0.40, 0.45 µg/ml). Each treatment was represented by four replicate plates. After incubation for 4 days at 25°C in darkness, colony diameter was measured at perpendicular angles, and the average of the two measurements (minus 5 mm for the mycelial plug) was used for data analysis. The percentage of inhibition was calculated for each concentration and the concentration of each fungicide causing 50% inhibition (EC_50_) was estimated from the regression of the probit of the percentage of growth inhibition against the logarithmic value of fungicide concentration. For each of the three CAA fungicides, the frequency distribution of 80 EC_50_ values was plotted as a representation of baseline sensitivity.

### Development of CAA-resistant Mutants of *P. melonis in vitro*

**RAPD.** To select *P. melonis* isolates with different genetic background for generation of CAA-resistant mutants, 15 isolates collected from different fields were randomly chosen for genetic relationship analysis by using RAPD, and one isolate of *P. drechsleri* was used as the outgroup control (Table 2). Mycelia were frozen in liquid nitrogen and ground into fine powder with mortar and pestle, which has been previously sterilized at 160°C for 2 h. Genomic DNA was extracted according to the modified Ristaino’s CTAB protocol [39]. About 100 mg of mycelial powder was placed in a 1.5-ml centrifuge tube. A 150-µl volume of extraction buffer (0.35 M sorbitol, 0.1 M Tris, 0.005 M EDTA [pH 7.5], and 0.02 M sodium bisulfite) was added, and the tube was then mixed with a vortex mixer. A 150-µl volume of nuclear lysis buffer (0.2 M Tris, 0.05 M EDTA [pH 7.5], 2.0 M NaCl and 2% CTAB [pH 7.5]) and 60 µl of 20% SDS (20 g SDS per 100 ml water) was added, and the tube was mixed again. After incubation at 65°C for 30 min, an isopyknic mixture of chloroform-isoamyl alcohol (24∶1, v/v) was added, and the tube was centrifuged for 15 min at 13,000 *g*. The aqueous phase was transferred to a new tube, and the chloroform extraction was repeated. After adding 0.1 volume of 3 M sodium acetate (pH 8.0) and 0.6 volume of cold isopropyl alcohol, DNA was precipitated at −20°C for 2 h. The tube was centrifuged at 13,000 *g* for 15 min, and the precipitate was washed with 75% ethanol and then dried at room temperature. DNA was resuspended using 50 µl of TE buffer (10 mM Tris-HCl, 0.1 mM EDTA [pH 8.0]) for PCR.

RAPD-PCR was performed with each of 16 decamer primers (Table 1). The primers were synthesized by Beijing Sunbiotech Co. Ltd. (Beijing, China). PCR was performed in a 25-µl volume containing 50 ng of template DNA, 1 µl of primer (10 µM), 2 µl of dNTP mixture (2.5 mM of each dNTP and 20 mM Mg^2+^), 2.5 µl of Easy Taq DNA Polymerase Buffer (10×), and 2.5 U of EasyTaq DNA Polymerse (TransGen Biotech, Beijing, China). Amplification was performed in a MyCycler™ Thermal Cycler (Bio-Rad) with the following parameters: 94°C for 6 min; followed by 40 cycles of denaturation at 94°C for 30 s, annealing at 36°C for 1 min, and extension at 72°C for 2 min and a final cycle of extension at 72°C for 10 min. Amplification products were separated on 1.5% agarose gels in Tris-acetate (TAE) buffer at 110 V for 2 h and were visualized under UV light after being stained with ethidium bromide. All PCRs were repeated at least twice.

Differences in fingerprinting patterns among isolates were assessed based on the clear and reproducible bands. Presumed homologous bands were scored zero (absent) or one (present) and then transformed into a binary matrix. Genetic distance coefficients were calculated for all pairwise comparisons by Nei’s method [40]. The phylogenic tree was generated based on the genetic distance coefficients by using UPGMA (unweighted pair-group method arithmetic averages) and MEGA (molecular evolutionary genetics analysis) software (version 5).

#### Generation of CAA-resistant isolates

Based on the genetic analysis, seven isolates of *P. melonis* were selected for generation of CAA-resistant mutants. In the case of flumorph, zoospores suspensions were prepared as described above, and 100 µl of a zoospore suspension (approximately 1.0×10^6^ zoospores/ml) was inoculated onto WKB plates amended with 10 µg/ml of flumorph (WKBF). After incubation at 25°C in darkness for 5 days, the emergent colonies were transferred to a fresh WKBF plate. Single-zoospore isolates were obtained. The same procedure was used for generation of resistant mutants to dimethomorph (10 µg/ml of medium) and iprovalicarb (5 µg/ml of medium). This selection procedure was performed twice.

#### Ultraviolet (UV)-mutagenesis of zoospores

Zoospore suspensions were continuously agitated while they were exposed to UV irradiation (TUV Philips, 15 W, 254 nm) for 1 min at a distance of 30 cm. The suspensions were then spread on WKB medium plates amended with the corresponding CAA fungicide as described in the previous section. These plates were incubated in the dark for 30 min to minimize light repair of DNA damage. This selection procedure was performed twice and included control plates that were not exposed to UV.

### Biological Characteristics of CAA-resistant Mutants

#### Stability and level of resistance

For determination of the stability of CAA resistance of the mutants, the mutants were subjected to 10 successive transfers on fungicide-free medium before mycelium growth was measured on WKB agar medium amended with each corresponding fungicide at the concentrations described previously. The experiment was done twice. EC_50_ values of mutants were estimated by measuring mycelium growth on fungicide-amended medium at 0, 5, 10, 20, 40, 80 and 100 µg/ml of each CAA fungicide. The level of resistance was described by the resistance factor: RF  =  EC_50_ of mutant at the 10^th^ transfer/EC_50_ of its parent.

In addition, one spontaneous and one UV-induced mutant resistant to each of fungicide was randomly selected for determination of resistance stability of zoospore progeny. At least 20 single-zoospore isolates randomly sampled from each mutant were grown on WKBF medium.If these single-zoospore isolates grew on WKBF medium, their resistance was considered to be stable; otherwise their resistance was unstable. The same procedure was followed with dimethomorph at 10 µg/ml and with iprovalicarb at 5 µg/ml. This experiment was performed twice.

#### Mycelial growth and zoospores production

For determination of mycelial growth, the 26 CAA-resistant isolates and their parents were transferred to WKB medium in plates as described in the section concerning baseline sensitivities except that the medium did not contain fungicide. Each isolate was represented by three replicated plates. After incubation in the darkness at 25°C for 5 days, the colony diameter was measured at perpendicular angles, and the average of the two measurements was used to compare the mycelial growth of each resistant isolate and its parent. For comparison of zoospore production, 10 plugs (5-mm in diameter) from the colony margin and 10 plugs from the area near the initial inoculum plug were harvested, and zoospore production was induced as described above and quantified with a hemacytometer. The number of zoospores per cm^2^ of culture was calculated. These experiments were conducted at least twice.

#### Virulence

The second or third true leaf from a cucumber plant (cv. Changchunmici) at the fifth true leaf stage was used for virulence tests. The leaves were harvested and rinsed three times with sterile-distilled water. Zoospore suspensions were prepared as described earlier for each of the 26 CAA-resistant mutants and their parent isolates. Four 10-µl droplets of a zoospore suspension (1.0×10^5^ zoospores/ml) were placed on the upper surface of leaves. One half of each leaf was inoculated with a resistant mutant and the other half was inoculated with the corresponding parent. Ten replicate leaves were used for each combination of mutant and parent. After 5 days in a moist chamber at 20°C with 12 h of light and 12 h of darkness, the lesion areas on each leaf were measured. This experiment was conducted at least twice for each combination of mutant and parent.

#### Cross-resistance

The 55 CAA-resistant mutants and 20 wild-type isolates were cultured on WKB agar medium amended with the non-CAA fungicides metalaxyl (0, 0.01, 0.02, 0.05, 0.10, and 0.20 µg/ml). azoxystrobin (0, 0.01, 0.05, 0.10, 0.50, and 1.00 µg/ml), cymoxanil (0, 10, 20, 40, 80, and 100 µg/ml), or cyazofamid (0.01, 0.02, 0.05, 1.00, and 2.00 µg/ml) or with the CAA fungicides at the concentrations described above. After incubation in darkness at 25°C for 4 days, the colony diameters were measured and the EC_50_ values were calculated as described above. Each treatment was represented by three replicate plates. The experiment was conducted at least twice for each isolate.

### Amplification of the *CesA*3 gene of *P. melonis*

Based on the conserved sequence of the *CesA3* genes in *P. infestans* (ABP96904), *P. ramorum* (ABP96912) and *P. sojae* (ABP96908) in the Genbank/EMBL data libraries, homologous primers were designed for amplification of the partial *PmCesA3* gene fragment. The 5′and 3′end of the *PmCesA3* gene were acquired using SiteFinding-PCR [41]. The full-length *PmCesA3* gene was amplified and sequenced using primers listed in Table 1. All primers were synthesized by Beijing Sunbiotech Co. Ltd. (Beijing, China). Primers PmA3F1 and PmA3R1 were used to amplify the *PmCesA3* gene. PCRs were performed in a 50-µl volume containing 50 ng of template DNA, 1 µl of each primer (10 µM), 4 µl of dNTP mixture (2.5 mM each dNTP), 1×Easy Taq DNA Polymerase Buffer, and 2.5 U of EasyTaq DNA Polymerase (TransGen Biotech, Beijing, China). The PCR was performed in a MyCycler™ Thermal Cycler (Bio-Rad) with the following parameters: an initial preheating for 5 min at 95°C; followed by 35 cycles of denaturation at 94°C for 30 s, annealing at 62°C for 30 s, and extension at 72°C for 4 min; and with a final extension at 72°C for 10 min. All PCR products were separated and purified by electrophoresis in a 1% agarose gel in Tris-acetate (TAE) buffer and were cloned into the pEASY-T3 Vector (TransGen Biotech, Beijing, China) and sequenced by Beijing Sunbiotech Co. Ltd. (Beijing, China). The programs in the DNAMAN software were used to predict the PmCesA3 amino acid sequences and to compare the amino acid sequences of the wild-type isolates with those of the CAA-resistant mutants.

### Molecular Detection of Resistance Mutation in PmCesA3 by Allele-specific PCR

According to the single mutation in the *PmCesA3* gene, allele-specific primers were designed with the match the nucleotide ‘C’ at the 3′-end of the reverse primers. The specificity of the primers was improved by introducing an artificial mismatch base at the second nucleotide at the 3′-end of the primers (Table 1). To test the specificity of the primers, all the primer pairs were used for gradient PCR using the DNA templates from wild-type isolate TJ-58 and CAA-resistant mutant F58-4. PCR amplification was performed in a MyCycler™ Thermal Cycler (Bio-Rad) with the following parameters: an initial preheating for 5 min at 95°C; followed by 30 cycles of denaturation at 95°C for 30 s, annealing at 50 to 70°C for 30 s, and extension at 72°C for 30 s; and terminated with a final extension at 72°C for 10 min. A 5-µl volume of PCR product from each sample was analyzed by electrophoresis using a 1.5% agarose gel in TAE buffer.

### Statistical Analysis

Data were analyzed by using the general linear model (GLM) procedure with Statistical Analysis System software (version 9; SAS Inc., Cary, NC, USA). Means were separated using Fisher’s protected least significant difference (LSD, α = 0.05). Cross-resistance between fungicides was analyzed using Spearman’s rank correlation coefficient for log-transformed EC_50_ values.
